# Coumarin–Ferulic Acid Hybrid Mediates HT-29-Selective Growth Inhibition and Proteomic Profiling

**DOI:** 10.3390/molecules31203729

**Published:** 2026-10-17

**Authors:** Sucheewin Krobthong, Meng Chieh Yang, Pattarawit Rukthong, Satsawat Visansirikul, Kittisak Sripha, Arnatchai Maiuthed

**Affiliations:** 1 Centre of Biopharmaceutical Science for Healthy Ageing, Faculty of Pharmacy, Mahidol University, Bangkok 10400, Thailand; sucheewin82@gmail.com (S.K.); mengchiehyang99@gmail.com (M.C.Y.); 2 Department of Pharmaceutical Technology, Faculty of Pharmacy, Srinakharinwirot University, Ongkharak, Nakhonnayok 25120, Thailand; pattarawit@g.swu.ac.th; 3 Center for Excellence in Plant and Herbal Innovation Research, Strategic Wisdom and Research Institute, Srinakharinwirot University, Nakornnayok 26120, Thailand; 4 Department of Pharmaceutical Chemistry, Faculty of Pharmacy, Mahidol University, Si Ayutthaya Rd, Bangkok 10400, Thailand; satsawat.vis@mahidol.ac.th (S.V.); kittisak.srp@mahidol.ac.th (K.S.); 5 Unit of Compound Library for Drug Discovery, Mahidol University, Bangkok 10400, Thailand; 6 Department of Pharmacology, Faculty of Pharmacy, Mahidol University, Bangkok 10400, Thailand

**Keywords:** coumarin hybrid, colorectal carcinoma, HT-29, structure activity relationship, quantitative proteomics, unfolded protein response

## Abstract

Coumarin-based molecular hybrids provide a framework for examining how structural changes influence cellular responses. This study compared four hybrids and three reference compounds across ten human cancer cell lines, with the lead hybrid also tested in non-cancerous Beas-2B cells, followed by functional assays and quantitative expression proteomics in HT-29 cells. Of 70 compound–cell-line combinations, the ferulic ester hybrid (FEC) was the only compound to yield a reproducibly measurable IC_50_ within the primary screening range, reaching 42.8 ± 4.7 µM in HT-29 cells. Replacing the ferulic moiety or the ester linkage reduced activity within this series. HCT116 cells did not reach 50% inhibition at up to 100 µM. Basal PPARγ abundance did not consistently explain sensitivity across the panel. FEC reduced metabolic activity and colony formation, whereas annexin V/propidium iodide analysis showed a smaller apoptotic response. Proteomics identified coordinated decreases in DNA replication and translation proteins and a modest increase in endoplasmic reticulum protein-folding enzymes. These findings support a structure-dependent and cell-context-dependent antiproliferative response despite moderate potency.

## 1. Introduction

Colorectal cancer is the third most frequently diagnosed malignancy and the second leading cause of cancer death worldwide [1]. Estimates based on GLOBOCAN 2022 place the lifetime risks of developing and dying from colorectal cancer at 2.69% and 1.39%, respectively, with substantial variation across countries and levels of development [2]. Fluoropyrimidines remain the backbone of systemic treatment for metastatic colorectal cancer, although toxicities such as cardiotoxicity and hand–foot syndrome may require treatment modification [1,3]. In advanced disease, fluoropyrimidines are combined with oxaliplatin or irinotecan, often together with an anti-angiogenic agent such as bevacizumab or, after oxaliplatin failure, Ziv-aflibercept [4,5,6]. Consequently, there remains a need for agents that act through mechanisms distinct from those of antimetabolite chemotherapy and that exhibit selective activity against biologically distinct tumors arising within the same tissue.

Natural products supply much of the chemical diversity behind such agents. Coumarins (2*H*-1-benzopyran-2-one or 1-benzopyran-2-one) are widely found in plants, and its derivatives display antioxidant, anti-inflammatory, antimicrobial and antineoplastic activities [7]. Therapeutic activity within the class depends heavily on the substitution pattern rather than on the benzopyrone core structure itself, and unsubstituted coumarin is metabolically liable to toxic products [8]. Ferulic acid (4-hydroxy-3-methoxycinnamic acid), a hydroxycinnamic acid abundant in cereal brans, has documented antiproliferative activity in colorectal models, although poor aqueous solubility limits its potency; formulation into polymeric micelles improves the half maximal inhibitory concentration in Caco-2 cells more than tenfold, with a range approximately 191 to 17 µg/mL [9,10]. Although both scaffolds possess pharmacologically relevant properties, their individual anticancer activity is weak.

Molecular hybridization offers a strategy for overcoming such limitations by covalently combining two pharmacophores within a single molecular entity. This approach aims to enhance biological activity, selectivity, or pharmacokinetic behavior that cannot be achieved by either parent compound alone [11]. The hybridization approach has generated bioactive series across many heterocyclic classes, and structure activity analyses consistently identify linker chemistry and substituent electronics as decisive for potency and selectivity [11,12]. For coumarin conjugates specifically, the biological consequences of linker choice have not been compared systematically within a matched series tested across a common cell panel.

The rationale for combining coumarin and ferulic acid is further supported by evidence linking both scaffolds to the regulation of protein homeostasis. Ferulic acid inhibits all three peptidase activities of the 26S proteasome in a concentration-dependent manner, including the human enzyme [13]. Coumarin-containing extracts preferentially inhibit the trypsin-like activity and dock to the β2 and β5 subunits of the 20S catalytic core [14], and psoralen-based furocoumarins have been developed deliberately as catalytic subunit inhibitors of the immunoproteasome [15]. Coumarin derivatives have also been reported to act indirectly on protein turnover, for example by promoting proteasomal degradation of β-catenin in bladder carcinoma cells [11,16]. Perturbation of proteostasis therefore represents a plausible, although unconfirmed, mechanistic link between these two pharmacophores. In addition, a complementary rationale for investigating coumarin hybrids in colorectal cancer involves peroxisome proliferator-activated receptor gamma (PPARγ). It is expressed in both colonic mucosa and colon-derived tumors, and its ligands can modulate cellular differentiation and apoptosis [17]. Whether the receptor acts as an oncogene or a tumor suppressor in this tissue is unresolved: troglitazone, a synthetic agonist, induces colonic tumors in wild type C57BL/6J mice and accelerates carcinogenesis in Apc1638N/+ Mlh1+/− animals (Adenomatous polyposis coli), indicating that receptor activation is not uniformly protective [18]. Consequently, changes in PPARγ abundance alone are insufficient to establish that a compound acts through this receptor, and additional mechanistic evidence is required. The study was designed to determine how the incorporated pharmacophores and linker chemistry influence antiproliferative activity and differential sensitivity across cell lines, with particular attention to colorectal cancer models. We further examined whether PPARγ expression was associated with compound sensitivity and used complementary viability, membrane-integrity, and clonogenic assays to distinguish growth-inhibitory from cytotoxic effects. Quantitative expression proteomics was employed to investigate the cellular pathways affected by treatment. The analysis was intended to define the structure–activity relationships of the coumarin hybrids and provide a framework for investigating their cellular mechanism of action.

## 2. Results

### 2.1. Cytotoxicity Screening of the Parent Compounds

**Figure 1:**
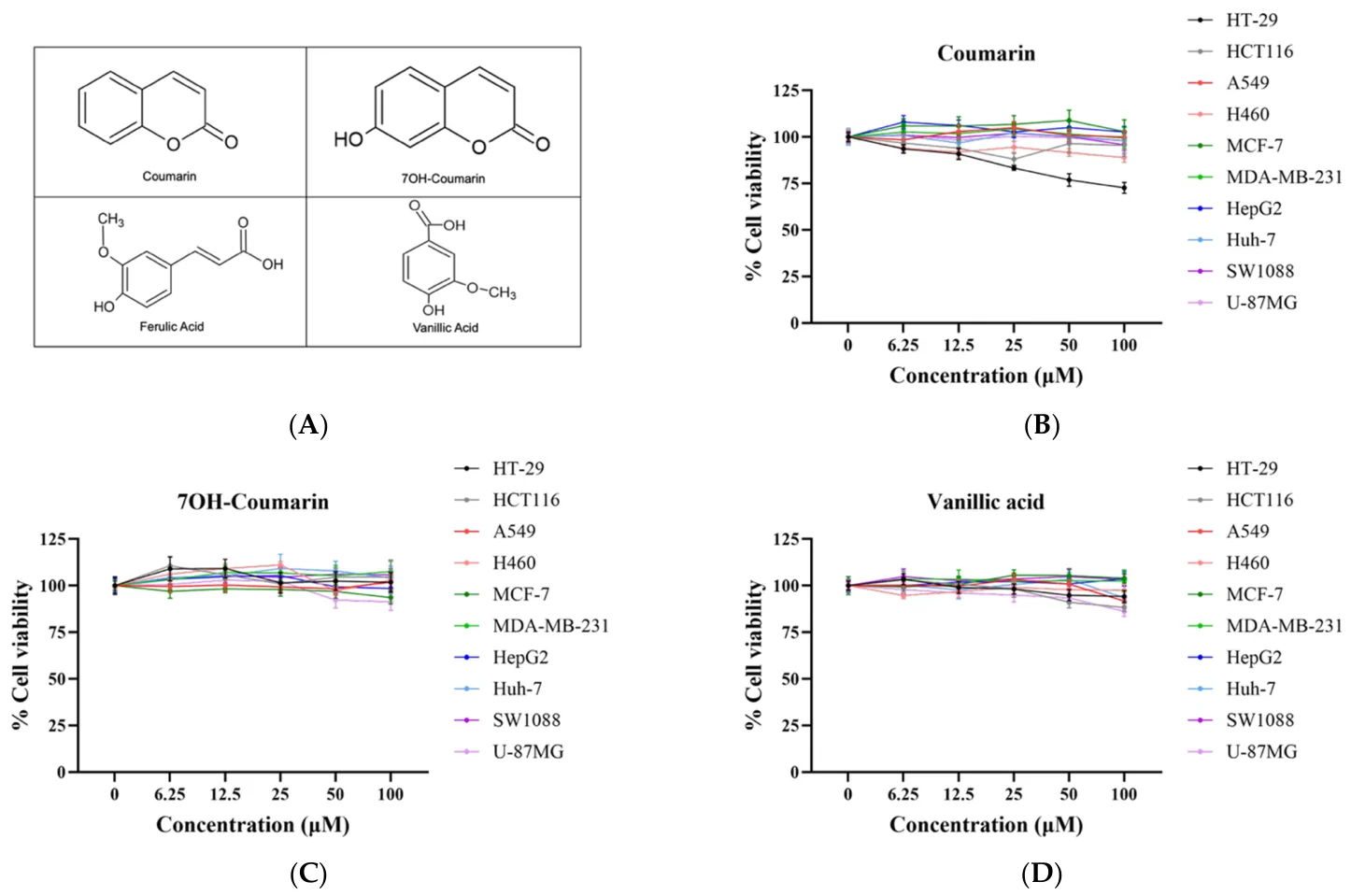
Chemical structures and cytotoxicity of the parent compounds. (**A**) Structures of coumarin, 7-hydroxycoumarin, ferulic acid and vanillic acid. (**B**–**D**) Concentration response curves for coumarin (**B**), 7-hydroxycoumarin (**C**) and vanillic acid (**D**) in SW1088, U-87MG, MCF-7, MDA-MB-231, A549, H460, HepG2, Huh-7, HCT116 and HT-29 cells treated for 24 h. Viability is expressed as a percentage of vehicle control. Data are mean ± SD of three independent experiments (four technical replicate wells per concentration in each experiment).

Coumarin, 7-hydroxycoumarin and vanillic acid were tested against all ten cell lines at concentrations up to 100 µM for 24 h. None reduced the MTT signal below 50% of the vehicle control. At 100 µM, residual MTT signals ranged from 72.7% to 102.9% for coumarin, 91.3% to 107.6% for 7-hydroxycoumarin, and 86.2% to 104.0% for vanillic acid. IC_50_ values for all 30 reference-compound–cell-line combinations were therefore reported as >100 µM. Ferulic acid is shown as a structural component in Figure 1A but was not included in this screening dataset.

### 2.2. Cytotoxicity of the Coumarin Hybrids Across the Cell-Line Panel

**Figure 2:**
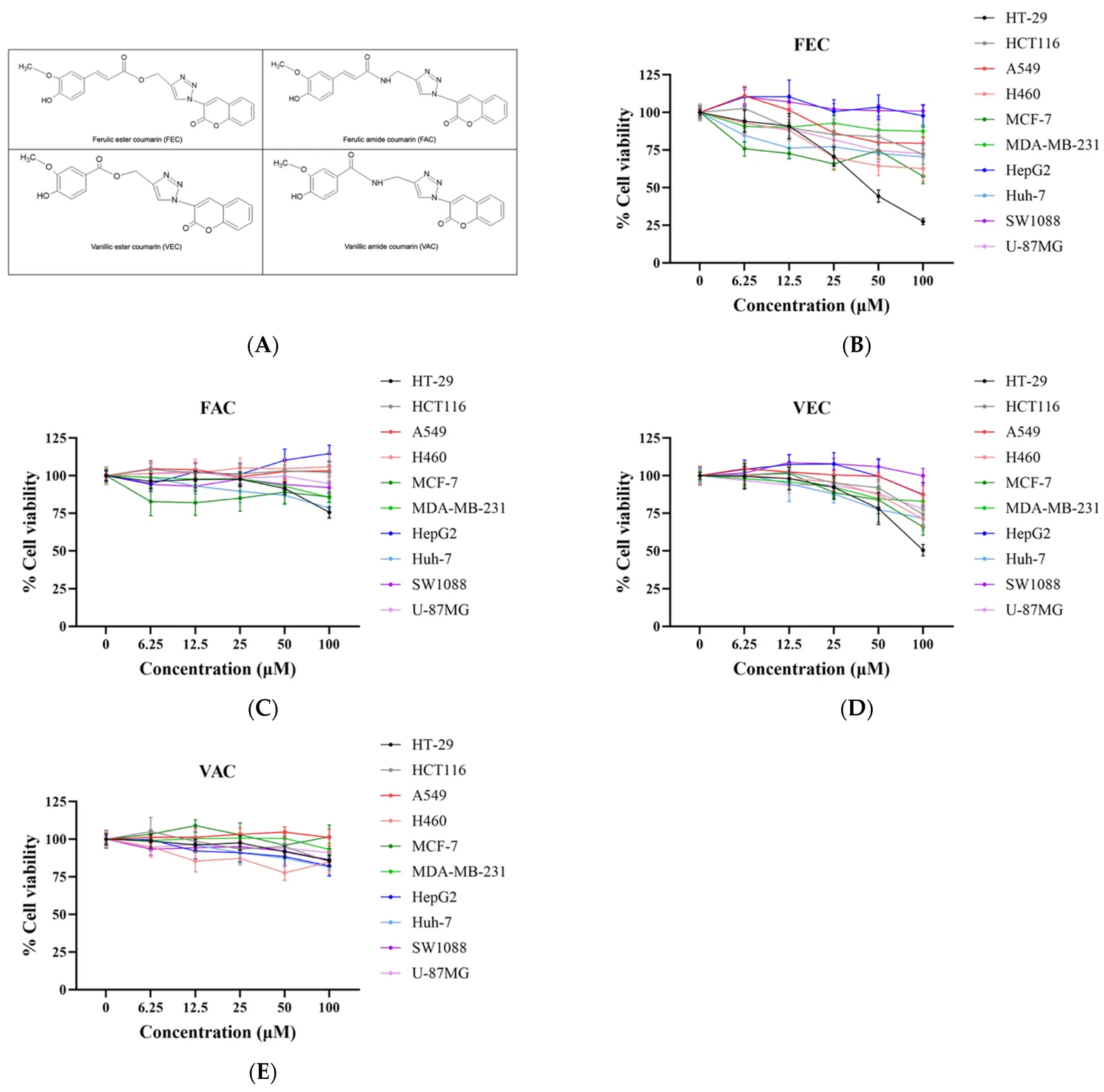
Structures and cytotoxicity of the coumarin hybrids. (**A**) Structures of FEC, FAC, VEC and VAC. (**B**–**E**) Concentration response curves for FEC (**B**), FAC (**C**), VEC (**D**) and VAC (**E**) across the ten cells panel after 24 h treatment. Data are mean ± SD of three independent experiments; versus vehicle control (one-way ANOVA with Dunnett’s post hoc test; four technical replicate wells per concentration in each experiment).

The four hybrids, ferulic ester coumarin (FEC), ferulic amide coumarin (FAC), vanillic ester coumarin (VEC) and vanillic amide coumarin (VAC), were tested against the same panel under identical conditions. Among forty hybrid–cell line combinations (four hybrids × ten cell lines), one produced a half-maximal inhibitory concentration within the tested range: FEC in HT-29 cells, at 42.8 ± 4.7 µM (n = 3 independent sets). VEC in HT-29 cells reached 50% inhibition in one of three sets only, with 48.6% viability at 100 µM in that set against 51.1% and 51.6% in the other two, and is therefore reported as marginal at approximately 100 µM rather than as a determined value. All remaining combinations were censored at >100 µM (see Figure 2).

Residual viability at 100 µM separated the series in HT-29 cells: FEC 27.4%, VEC 50.4%, FAC 75.7% and VAC 85.9%, compared with 72.7% for coumarin, 94.2% for vanillic acid and 101.8% for 7-hydroxycoumarin. Across the panel, FEC produced its lowest residual viability in HT-29 cells (27.4%), followed by MCF-7 (57.5%), H460 (62.5%), Huh-7 (70.4%), HCT116 (72.2%), U-87MG (72.6%), A549 (79.4%), MDA-MB-231 (87.4%), HepG2 (97.8%) and SW1088 (100.9%). In the extended concentration response experiment, FEC reduced HT-29 viability to 100, 100, 102, 97, 92, 64, 35, 30, 26 and 23% at 0, 1.95, 3.91, 7.81, 15.63, 31.25, 62.5, 125, 250 and 500 µM, respectively. Statistical significance was first reached at 15.63 µM, and *p* < 0.0001 from 31.25 µM upward.

Three further comparisons were run under the same 24 h MTT conditions (Figure S1). Ferulic acid, the head group of FEC, left HT-29 viability between 96.5% and 103.1% across 6.25–100 µM (IC_50_ > 100 µM), so the free acid does not reproduce the activity of the hybrid. Doxorubicin, included as a clinical positive control, reduced HT-29 viability to 75.4%, 67.3%, 58.1%, 46.7% and 39.2% at 6.25, 12.5, 25, 50 and 100 µM (*p* < 0.0001 at every concentration), corresponding to an IC_50_ of approximately 44.24 µM. In the non-cancerous bronchial epithelial line Beas-2B, FEC left viability at 97.5%, 94.8%, 92.7%, 95.0% and 103.1% over the same range (IC_50_ > 100 µM), with a small but significant decrease at 25 µM only. The selectivity index of FEC (IC_50_ in Beas-2B/IC_50_ in HT-29) is therefore >2.3, and FEC and doxorubicin were of comparable potency in HT-29 cells under these conditions (see Table 1).

### 2.3. PPARγ Abundance and Its Relationship with FEC Response

**Figure 3:**
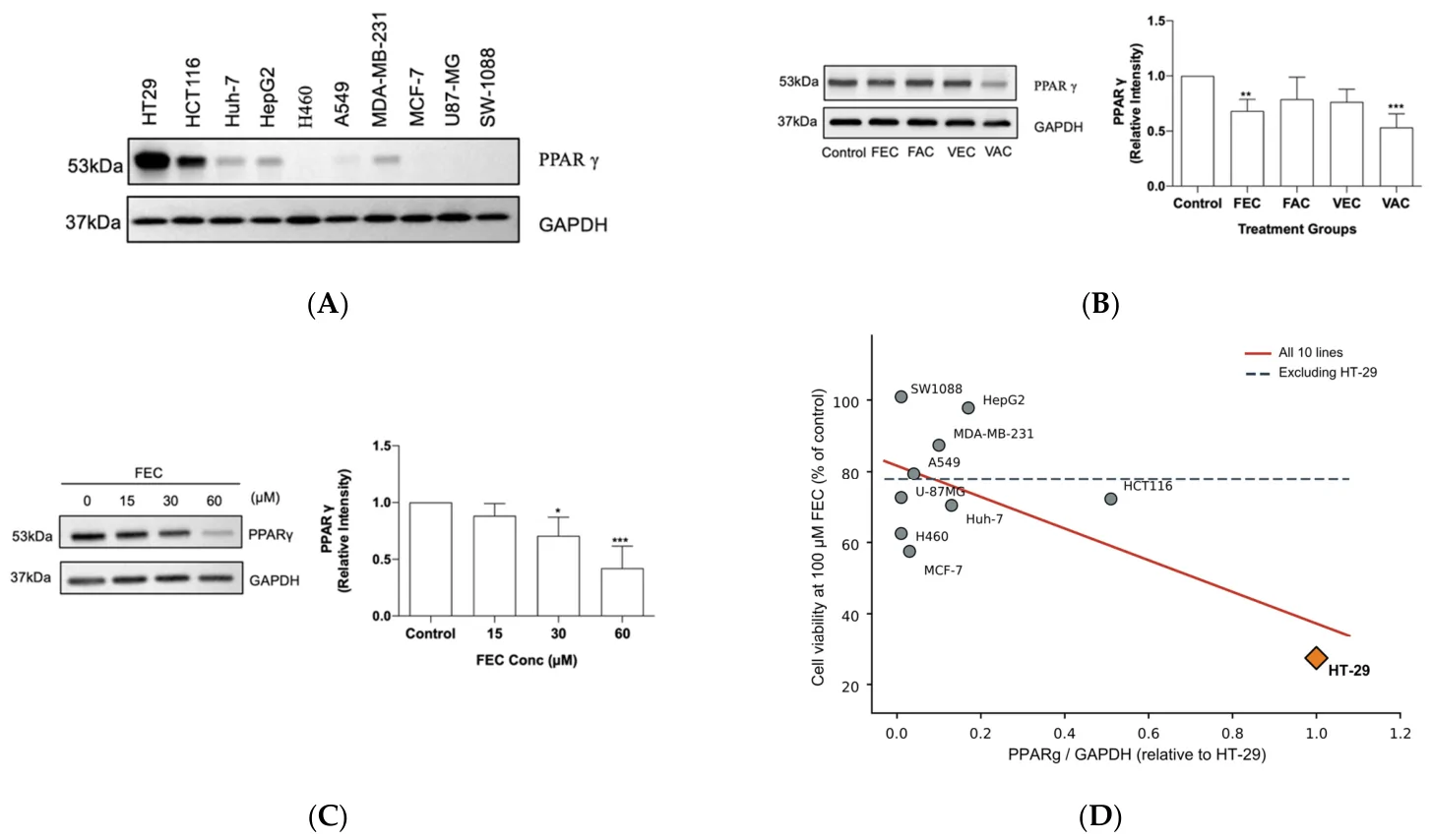
PPARγ expression and its relationship to FEC sensitivity. (**A**) Representative immunoblot of PPARγ across the ten-line panel, with GAPDH as loading control. (**B**) PPARγ after 24 h treatment of HT-29 cells with FEC, FAC, VEC or VAC. Mean ± SD of three independent experiments; one-way ANOVA with Dunnett’s post hoc test versus vehicle control; * *p* < 0.05, ** *p* < 0.01, *** *p* < 0.001. (**C**) PPARγ in HT-29 cells after 24 h treatment with 0, 15, 30 or 60 µM FEC. Mean ± SD of three independent experiments; one-way ANOVA with Dunnett’s post hoc test versus vehicle control. (**D**) Scatter plot of basal PPARγ to GAPDH ratio against residual viability at 100 µM FEC for the ten cell lines, with HT-29 highlighted. Solid line, regression through all ten cell lines; dashed line, regression excluding HT-29.

Baseline cellular PPARγ protein abundance was measured across the ten cell lines by Western blotting, with GAPDH used as the loading control. As shown in Figure 3A, the GAPDH signal was relatively uniform across the lanes, with a coefficient of variation of 13%. PPARγ was most abundant in HT-29 cells. After normalization to the PPARγ/GAPDH ratio in HT-29 cells, the relative values were 0.51 for HCT116, 0.17 for HepG2, 0.13 for Huh-7, 0.10 for MDA-MB-231, 0.04 for A549, 0.03 for MCF-7, and 0.01 for H460, U-87MG, and SW1088. However, PPARγ abundance did not consistently correspond with sensitivity to the treatment. HCT116 exhibited the second-highest PPARγ level but retained 72.2% viability, whereas MCF-7 was the second-most sensitive cell line, with 57.5% residual viability despite its low relative PPARγ level of 0.03. Similarly, HepG2 showed detectable PPARγ expression at 0.17 but was the least sensitive cell line tested, retaining 97.8% viability.

Treatment of HT-29 cells with the four hybrids for 24 h produced different PPARγ responses (Figure 3B). The plotted mean relative intensities were approximately 0.7 for FEC, 0.8 for FAC, 0.8 for VEC, and 0.5 for VAC, relative to the vehicle control. Figure 3B indicates significant decreases for FEC and VAC. In the FEC concentration–response experiment, the plotted means were approximately 0.9, 0.7, and 0.4 at 15, 30, and 60 µM, respectively, with significant decreases indicated at 30 and 60 µM (Figure 3C). These rounded values describe the displayed bar heights.

The association between basal PPARγ abundance and sensitivity to FEC was subsequently examined (Figure 3D). No robust association was found. Across all ten cell lines, the rank-based Spearman correlation between the PPARγ/GAPDH ratio and residual viability at 100 µM FEC was not significant (ρ = −0.227, *p* = 0.528). A nominally significant Pearson correlation (r = −0.661, *p* = 0.038) was driven by HT-29 as a high-leverage observation, as shown by leave-one-out analysis. After excluding HT-29, no association remained according to either Pearson (r = −0.002, *p* = 0.996) or Spearman (ρ = +0.068, *p* = 0.862) correlation analysis. Thus, the three counterexamples described above reflect the broader pattern among the remaining cell lines rather than isolated deviations from an otherwise consistent association.

### 2.4. Apoptosis and Colony Formation in FEC-Treated HT-29 Cells

**Figure 4:**
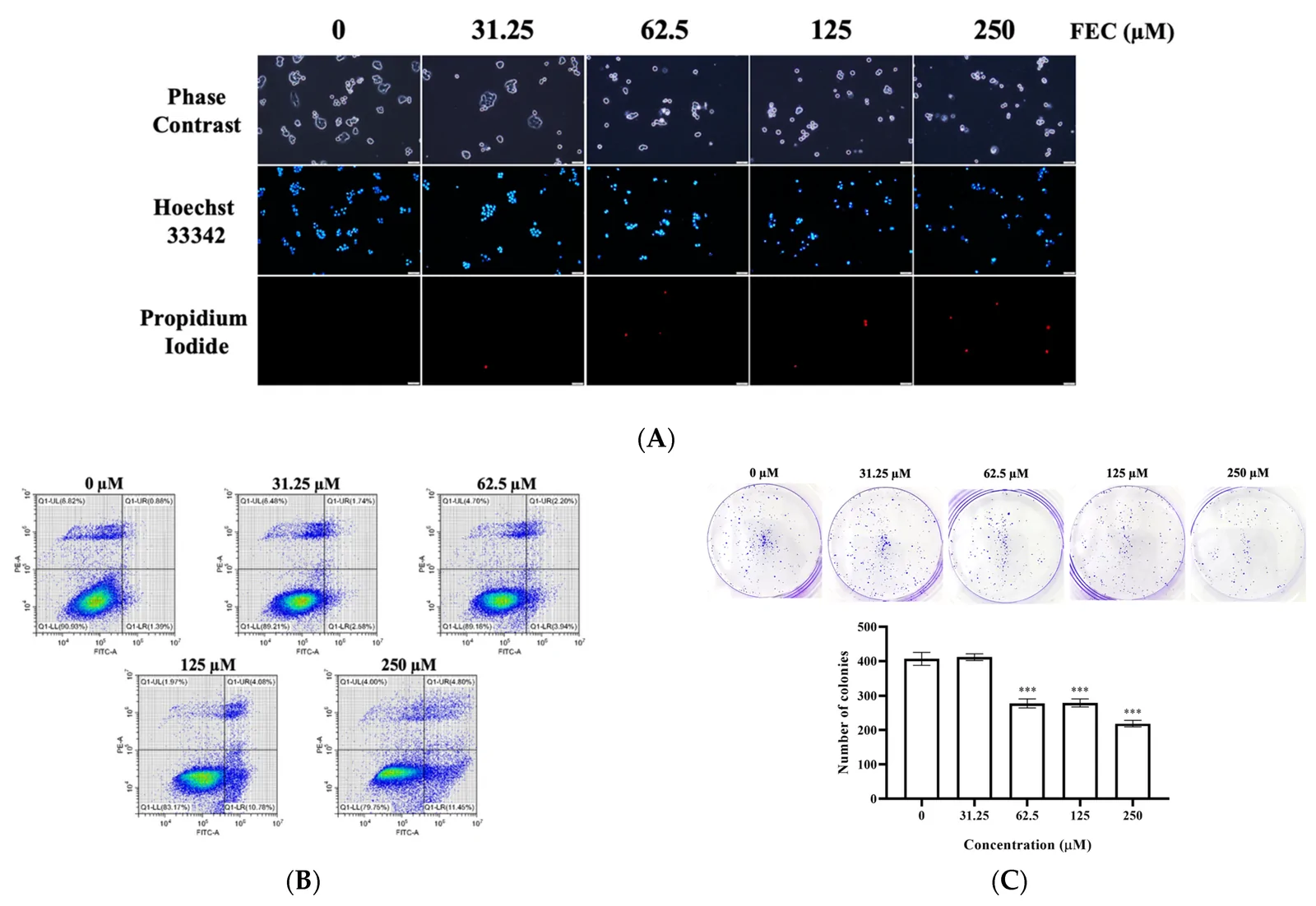
Cellular response of HT-29 cells to FEC. (**A**) Representative annexin V and propidium iodide dot plots at 0, 31.25, 62.5, 125 and 250 µM FEC, with quadrant percentages. (**B**) Apoptosis analysis of FEC-treated HT-29 cells (single experiment). (**C**) Representative crystal violet-stained wells and colony counts for the displayed doses of 0, 31.25, 62.5, 125, and 250 µM. Colony counts are mean ± SD of three independent experiments, each with triplicate wells; *** *p* < 0.001 versus vehicle control (one-way ANOVA with Dunnett’s post hoc test).

Phosphatidylserine externalization was assessed using annexin V and propidium iodide flow cytometry in a single experiment (Figure 4A), these values are therefore descriptive and were not tested statistically. The proportion of viable cells decreased from 90.93% in the vehicle control to 89.21%, 89.16%, 83.17%, and 79.75% following treatment with 31.25, 62.5, 125, and 250 µM FEC, respectively (Figure 4B). In parallel, the early apoptotic population increased from 1.39% in the vehicle control to 2.58%, 3.94%, 10.78%, and 11.45%, whereas the late apoptotic population increased from 0.86% to 1.74%, 2.20%, 4.08%, and 4.80%, respectively.

Consequently, the total apoptotic fraction increased from 2.25% in the vehicle control to 16.25% at 250 µM FEC. The proportion of necrotic cells showed no concentration-dependent trend, with values of 6.82%, 6.48%, 4.70%, 1.97%, and 4.00% for the vehicle control and increasing FEC concentrations, respectively.

Clonogenic survival was assessed following 24 h of FEC treatment and a subsequent 14-day colony-formation period (Figure 4C). Colony numbers were 406 ± 18, 414 ± 22, 277 ± 15, 279 ± 12, and 217 ± 9 at FEC concentrations of 0, 31.25, 62.5, 125, and 250 µM, respectively. A significant reduction in colony formation was observed from 62.5 µM onward (*p* < 0.001). Colony numbers were comparable between 62.5 and 125 µM, indicating no further reduction within this concentration range. The greatest effect occurred at 250 µM, where colony formation was reduced by 46.6% relative to the vehicle control. Because 2000 viable cells were replated per well, these counts correspond to plating efficiencies of 20.3%, 20.7%, 13.9%, 14.0% and 10.9%, and surviving fractions of 1.00, 1.02, 0.68, 0.69 and 0.53, respectively. The number of viable cells recovered after the 24 h expo-sure was not recorded; the assay therefore reflects the colony-forming capacity of surviving cells rather than total treatment-induced cell loss.

### 2.5. Proteomic Profiling of FEC-Treated HT-29 Cells

**Figure 5:**
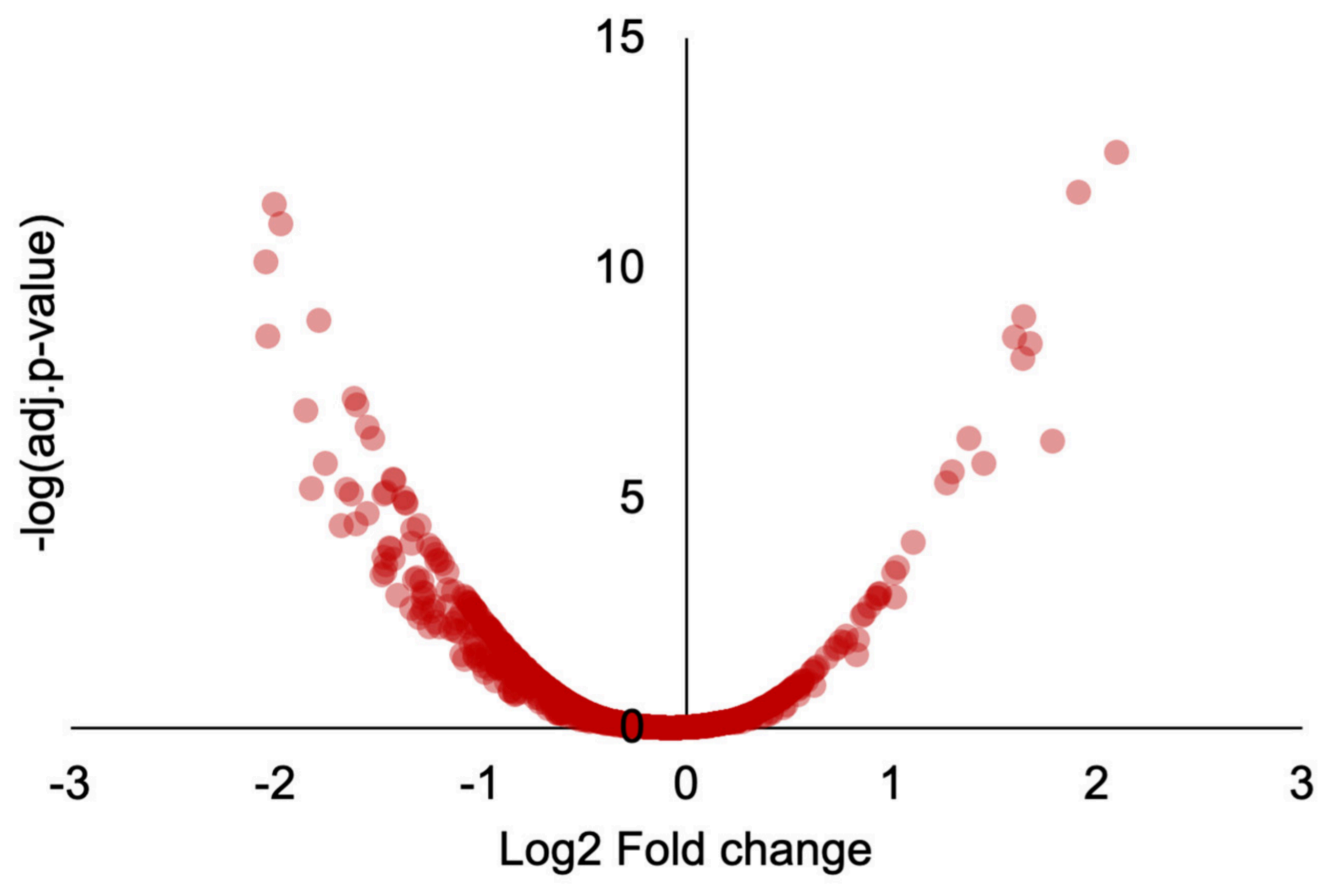
Proteomic response of HT-29 cells to FEC cells to FEC (n = 4 biological replicates per condition). Volcano plot of log_2_ fold change against −log_10_ adjusted *p* value for the 2398 analyzable proteins, with the 176 proteins passing an adjusted *p* value below 0.05 and an absolute log_2_ fold change of at least 1 highlighted and the fifteen strongest changes in each direction labelled.

Quantitative proteomic comparison of FEC-treated and vehicle-treated HT-29 cells identified 2655 proteins, of which 2549 had calculable abundance ratios. Among these, 151 proteins were assigned ratios at the software boundaries of 100 or 0.01, indicating detection in only one condition. These proteins were therefore classified separately as presence–absence changes: 61 were detected exclusively in FEC-treated samples, whereas 90 were confined to vehicle-treated controls. The remaining 2398 proteins constituted the analyzable dataset (Supplementary Table S1). Among these proteins, 245 showed significant differences at an adjusted *p*-value below 0.05, including 66 increases and 179 decreases. When an additional threshold of |log_2_ fold change| ≥ 1 was applied, 176 proteins remained, comprising 50 increases and 126 decreases. Thus, decreases in protein abundance occurred more than twice as frequently as increases (Figure 5).

The largest decreases were observed for ABHD11 (log_2_FC = −2.05, adjusted *p* = 7.4 × 10^−11^), PRIM2 (−2.04, 3.1 × 10^−9^), ACTBL2 (−2.01, 4.2 × 10^−12^), CDK1 (−1.97, 1.2 × 10^−11^), NUP50 (−1.85, 1.3 × 10^−7^), CUL5 (−1.83, 6.4 × 10^−6^), PTMS (−1.76, 1.8 × 10^−6^), ORC2 (−1.68, 4.1 × 10^−5^), PPP2R2D (−1.65, 6.5 × 10^−6^), TYMS (−1.63, 8.4 × 10^−6^), TNPO3 (−1.62, 6.9 × 10^−8^), LARP1 (−1.55, 3.0 × 10^−7^), LIG1 (−1.46, 2.9 × 10^−4^), H3-3A (−1.42, 4.2 × 10^−6^), and CLPTM1L (−1.33, 4.8 × 10^−5^). The clearest increases were detected for TPP1 (log_2_FC = +2.10, adjusted *p* = 3.2 × 10^−13^), NDUFB3 (+1.91, 2.3 × 10^−12^), PELO (+1.79, 6.0 × 10^−7^), MRPS33 (+1.68, 4.5 × 10^−9^), RAB6D (+1.64, 1.2 × 10^−9^), SPEN (+1.64, 9.3 × 10^−9^), ATP5PF (+1.60, 3.3 × 10^−9^), LARP4 (+1.45, 1.8 × 10^−6^), RBM34 (+1.38, 5.0 × 10^−7^), MGST2 (+1.30, 2.7 × 10^−6^), AIMP1 (+1.27, 4.8 × 10^−6^), and ACTG1 (+1.03, 3.2 × 10^−4^). Several of the most strongly decreased proteins, including PRIM2, ORC2, LIG1, CDK1, and TYMS, participate in DNA replication or nucleotide metabolism. In addition, LARP1 and LARP4 are involved in regulating the translation of transcripts encoding ribosomal proteins.

Competitive testing of curated protein sets against the remainder of the analyzable proteome, which had a median log_2_ fold change of −0.097, indicated that the proteomic response was coordinated rather than sporadic. DNA replication proteins showed the strongest collective decrease (n = 21, median = −0.344; q = 1.7 × 10^−3^), followed by translation factors (n = 37, median = −0.206; q = 0.040) and cytosolic ribosomal proteins (n = 73, median = −0.203; q = 7.3 × 10^−3^). Notably, 89% of the cytosolic ribosomal proteins changed in the negative direction. Conversely, two protein sets showed coordinated increases. Endoplasmic reticulum protein-folding enzymes showed a small collective shift towards higher abundance (n = 13, median = +0.104; q = 1.7 × 10^−3^), with every quantified member displaying a positive log_2_ fold change, whereas mitochondrial ribosomal proteins showed a marginal increase (n = 49, median = +0.057; q = 0.050). Within the endoplasmic reticulum protein-folding set, the largest individual changes were observed for HSPA5 (+0.30), SDF2L1 (+0.27), DNAJB11 (+0.21), and PDIA4 (+0.20). None of these proteins reached individual significance; therefore, evidence for alteration of this module was based on the coordinated direction of the complete protein set rather than any single component.

Four curated protein sets showed no significant collective change. The 20S proteasome catalytic core remained unchanged (n = 14, median = −0.084; q = 0.96), as did the cytosolic chaperone and heat-shock protein set (n = 37, median = −0.063; q = 0.50) and the spliceosome (n = 42, median = −0.159; q = 0.24). Subunits of the 19S proteasome regulatory particle showed a downward trend, although this change did not reach significance (n = 19, median = −0.181; q = 0.15). PPARG was not detected at the proteomic depth achieved, and neither were ATF4, DDIT3, XBP1, or HSF1.

## 3. Discussion

Coumarin and the hydroxycinnamic acids are individually weak antiproliferative agents, and the present results are consistent with that expectation, since none of the three parent compounds available to us reduced viability below half of control in any of ten cell lines. Covalent hybridization altered this outcome, but only under narrow structural conditions. Among seventy compound and cell line combinations, a single one, FEC against HT-29, produced a determinable IC_50_ of 42.8 ± 4.7 µM. Both the ferulic head group and the ester linkage appeared necessary, since replacing the head group with vanillic acid or the ester with an amide progressively reduced activity. Ferulic acid itself left HT-29 viability unchanged at up to 100 µM (Figure S1A), so the activity of FEC does not simply reflect the free acid and is gained through hybridization. Such fine dependence on structure argues against non-specific mechanisms such as membrane perturbation or redox cycling, which would be unlikely to discriminate between closely related analogues, and it accords with structure activity analyses across hybrid series in which linker design and substituent electronics govern potency and selectivity [12]. The activity is nonetheless modest in absolute terms, lying in the tens of micromolar, and FEC is best regarded as a lead requiring optimization rather than as a candidate compound.

Two reference compounds place this potency in context. Ferulic acid gave no inhibition of HT-29 cells at up to 100 µM here, consistent with published work in which the free acid inhibited Caco-2 cells only at about 191 µg/mL (roughly 980 µM) unless formulated into micelles [9]. Doxorubicin, measured in the same assay, gave an IC_50_ of approximately 44.24 µM, close to the 42.8 µM obtained for FEC. This does not make FEC equivalent to an established drug, because doxorubicin acts faster in longer exposures and has a defined clinical dose, but it does show that the 24 h endpoint used here places FEC within the same range as a widely used cytotoxic agent rather than orders of magnitude below it.

The ten-cell-line panel adds information about cellular context. HT-29 was the only line in which FEC yielded a reproducibly measurable IC_50_ within the primary screening range; HCT116 did not reach this endpoint despite sharing a colorectal origin. This contrast identifies a response difference for further investigation but does not establish a specific sensitivity determinant. Conventional IC_50_ and endpoint response measurements can be influenced by cell division rate and assay duration, as demonstrated by Hafner et al. [19]. Because growth-rate-normalized measurements were not obtained here, proliferation-related differences cannot be excluded. More importantly, all ten models were cancer cell lines. The observed pattern represents differential sensitivity among these cancer models. In non-cancerous Beas-2B cells, FEC left viability above 92% at up to 100 µM, an initial indication that normal epithelial cells tolerate concentrations that inhibit HT-29 cells. Beas-2B cells are bronchial rather than colonic, so this comparison shows general normal-cell tolerance rather than tissue-matched selectivity, and one normal line does not define a therapeutic window.

PPARγ was more abundant in HT-29 cells than in the other nine lines, and its abundance decreased with increasing FEC concentration. These observations initially suggested a possible relationship between PPARγ and FEC sensitivity, but analysis across the complete cell-line panel did not support this interpretation. Basal PPARγ abundance was not robustly associated with residual viability: the Spearman correlation was not significant, and a nominal Pearson correlation (r = −0.661, *p* = 0.038) depended entirely on HT-29, falling to −0.002 once this observation was removed. HCT116, MCF-7, and HepG2 illustrated the lack of a consistent relationship. Within the compound comparison, VAC produced the largest PPARγ decrease despite weaker growth inhibition. These results argue against using basal PPARγ abundance alone as a predictor of FEC sensitivity. They do not exclude a functional role for the receptor, which would require perturbation or target-engagement experiments. The context dependence of PPARγ-associated effects is also illustrated by the troglitazone findings in mouse colon tumor models [18].

Expression proteomics identified coordinated changes in several functional groups. DNA replication proteins showed the greatest decline (median log_2_FC = −0.344; q = 1.7 × 10^−3^), followed by translation factors (median log_2_FC = −0.206; q = 0.040) and cytosolic ribosomal proteins (median log_2_FC = −0.203; q = 7.3 × 10^−3^). Within the cytosolic ribosomal group, 89% of the 73 quantified proteins changed in the negative direction. Endoplasmic reticulum folding enzymes showed the opposite pattern, with all 13 quantified members displaying positive log_2_ fold changes (median = +0.104; q = 1.7 × 10^−3^). HSPA5, SDF2L1, DNAJB11, and PDIA4 showed the largest changes within this group. The relevant observation is thus a modest collective abundance shift, not demonstrated induction of each chaperone. GRP78/BiP participates in endoplasmic reticulum homeostasis and interactions with unfolded protein response sensors [20,21], making this pathway a reasonable hypothesis for follow-up. However, protein abundance changes do not measure folding activity or translation rate, and the absence of ATF4, DDIT3, and XBP1 from the dataset prevents assignment to a specific signaling branch. Under endoplasmic reticulum stress, PERK-mediated phosphorylation of eIF2α couples folding capacity to global protein synthesis [22,23]; whether FEC engages this branch was not tested.

Individual abundance changes were consistent with altered biosynthetic demand. PRIM2, CDK1, ORC2, TYMS, and LIG1 decreased, but these findings alone cannot establish cell-cycle arrest or its phase. LARP1, a regulator of terminal oligopyrimidine mRNA translation downstream of mTORC1 [24], also decreased. Its loss cannot by itself demonstrate translational suppression because its regulatory effects depend on functional state and context. Demonstrating reduced translation would require direct measurement of protein synthesis, for example by puromycin incorporation (SUnSET) or polysome profiling [25]. Increased PELO, which participates in ribosome rescue and translation initiation-site selection [26], provides another candidate for follow-up. PROM1/CD133 abundance decreased as well. Its roles have been examined in intestinal tumorigenesis and in stem-like leukemia populations [27,28], but a decrease in this marker does not establish elimination of colorectal cancer stem cells. These observations should guide functional testing rather than be treated as evidence that FEC directly targets any of these proteins.

The decrease in PPARγ after treatment may be part of a broader change in protein production or turnover. This interpretation is compatible with the expression-proteomics profile but remains untested. Neither receptor binding nor the rates of PPARγ synthesis and degradation were measured. Target-engagement assays, translation measurements, and protein-turnover experiments would be needed to distinguish these possibilities. Unchanged proteasome-subunit abundance likewise cannot exclude inhibition of proteasome activity.

The complementary cellular endpoints help interpret the response. At 250 µM FEC, the MTT signal was reduced by 74% and colony formation by 46.6%, whereas the apoptotic fraction in a single experiment was 16.25% and 79.75% of cells remained in the annexin V-negative/PI-negative population. These measurements assess different properties and should not be equated. MTT reduction depends on cellular metabolism and can be influenced by cell number, assay conditions, and chemical interference [29]. The smaller apoptotic response, together with reduced colony formation after compound withdrawal, supports an antiproliferative component that is not fully explained by acute apoptosis. It does not prove a purely cytostatic mechanism or exclude delayed cell death. The use of orthogonal endpoints is a strength, while direct proliferation and cell-cycle measurements remain necessary to resolve the phenotype.

Published ferulic acid and coumarin studies provide context rather than direct potency or safety benchmarks. Ferulic acid-loaded polymeric micelles produced antiproliferative and apoptotic effects in Caco-2 cells [9], and photoactivated trimethylangelicin produced apoptosis in bladder carcinoma cells [16]. Differences in compound identity, formulation, exposure, and cell model prevent quantitative transfer of these findings to FEC. FEC was originally synthesized and characterized as compound CE5 by Visansirikul et al., who studied it in a neuroprotection model and selected 2.5 µM as a non-toxic concentration in BV-2 cells after 24 h [30]. This concentration is below the range producing substantial HT-29 inhibition here, and BV-2 cells are murine microglia rather than normal human cells. That report therefore cannot establish FEC safety at anticancer concentrations. Similarly, reported tolerability of a parent scaffold or another hybrid cannot demonstrate low toxicity of this ester-linked derivative. The Beas-2B data obtained here give an initial selectivity index > 2.3, but testing normal colon epithelium, longer exposures and in vivo tolerability remains necessary before claims of tumor selectivity or safety can be made.

Taken together, the study contributes a paired comparison of head-group and linker changes, a ten-cell-line response profile, an assessment of the robustness of a proposed PPARγ association, and a proteomic map linked to complementary cellular endpoints. These findings retain value despite the compound’s moderate potency because they define which structural and cellular comparisons merit follow-up. The principal limitations are the use of a single, non-colonic normal cell line, the small analog series, the single annexin V experiment, and the absence of direct target and pathway validation. FEC is therefore best described as an exploratory compound for further chemical and mechanistic investigation, rather than a validated therapeutic lead.

## 4. Materials and Methods

### 4.1. Parent Compounds

The four coumarin hybrid molecules, ferulic ester coumarin (FEC), ferulic amide coumarin (FAC), vanillic ester coumarin (VEC) and vanillic amide coumarin (VAC), were kindly provided by Assoc. Prof. Kittisak Sripha (Department of Pharmaceutical Chemistry, Faculty of Pharmacy, Mahidol University, Thailand) [30]. These compounds correspond to CE5 (FEC), CA5 (FAC), CE4 (VEC) and CA4 (VAC) in that report, where their structures were confirmed by ^1^H and ^13^C NMR, FTIR and HRMS, and their purity was determined as 100% by HPLC (YMC-Pack Pro C18 column, Agilent system, Santa Clara, CA, USA) [25]. No new purity analysis was performed on the batches used here. Coumarin (Cat no. C4261, Sigma, St. Louis, MO, USA), 7-hydroxycoumarin, vanillic acid, ferulic acid and doxorubicin hydrochloride (Sigma Aldrich, St. Louis, MO, USA, cat. no. D1515) were obtained commercially. All compounds were dissolved in dimethyl sulfoxide (DMSO) and diluted in culture medium immediately before use, with the final DMSO concentration held below 0.1% (*v*/*v*) in all treatments and in the vehicle control. Wells were inspected under an inverted microscope during treatment, and no precipitate or turbidity was observed at any concentration used, including 500 µM. Concentrations of 31.25–250 µM, approximately 0.7–6 times the 24 h MTT IC_50_, were used in the mechanistic assays to span the partial-to-maximal range of the FEC concentration–response curve, which approached a plateau above 125 µM.

### 4.2. Cell Culture and Cell Viability Assay

Ten human cancer cell lines were used: SW1088 and U-87MG (brain), MCF-7 and MDA-MB-231 (breast), A549 and H460 (lung), HepG2 and Huh-7 (liver), and HCT116 and HT-29 (colorectal). The cell lines were obtained from the American Type Culture Collection (ATCC, Manassas, VA, USA). SW1088, A549, U-87MG, MDA-MB-231, HCT116, and HT-29 were maintained in Dulbecco’s modified Eagle’s medium (DMEM); Huh-7 was maintained in low-glucose DMEM; and MCF-7, H460, and HepG2 were maintained in RPMI 1640 medium (Gibco, Invitrogen, Melbourne, Australia). Media contained 10% fetal bovine serum and penicillin–streptomycin. Cells were incubated at 37 °C in 5% CO_2_. The non-cancerous human cell line Beas-2B (ATCC CRL-9609) was maintained in Bronchial Epithelial Growth Medium with 10% fetal bovine serum and penicillin–streptomycin.

Cell cytotoxicity was assessed using the MTT assay [31]. Huh-7, HepG2, and MCF-7 cells were seeded at 2 × 10^4^ cells per well, and the remaining cell lines at 1 × 10^4^ cells per well, in 96-well plates. After 24 h, cells were exposed to the test compounds at 0, 6.25, 12.5, 25, 50, or 100 µM for a further 24 h. An additional FEC concentration–response experiment in HT-29 cells used 0, 1.95, 3.91, 7.81, 15.63, 31.25, 62.5, 125, 250, and 500 µM. FEC was additionally tested in Beas-2B cells, and ferulic acid and doxorubicin in HT-29 cells, over the same 0–100 µM range. Each concentration was tested in four replicate wells in each of three independent experiments. The medium was then removed, and 100 µL of MTT solution (0.2 mg/mL in PBS) was added per well. After 3 h at 37 °C, the MTT solution was replaced with 100 µL of DMSO per well, and absorbance was measured at 570 nm using a microplate reader. The MTT signal relative to the vehicle control was calculated as follows:

Cell viability (%) = Mean OD_570_/Control OD_570_ × 100%

OD_570_ denotes absorbance at 570 nm. Signals were normalized to vehicle-control wells on the same plate. IC_50_ was defined as the concentration associated with a 50% reduction in the MTT signal relative to the vehicle control. Values were reported as >100 µM when the primary screen did not reach this endpoint. These values describe the endpoint MTT response and are not growth-rate-normalized inhibition estimates. The selectivity index was calculated as the IC_50_ in Beas-2B cells divided by the IC_50_ in HT-29 cells.

### 4.3. Annexin V and Propidium Iodide Flow Cytometry

HT-29 cells were seeded in 6-well plates at a density of 5 × 10^5^ cells per well and maintained at 37 °C in 5% CO_2_ for 24 h. The cells were then treated with 0, 31.25, 62.5, 125, or 250 µM FEC for 24 h. Cells were harvested by trypsinization and subjected to apoptosis analysis using the Alexa Fluor^®^ 488 Annexin V/Dead Cell Apoptosis Kit (Invitrogen, Cat. #V13241) in accordance with the manufacturer’s protocol [32]. Briefly, 1 × 10^6^ cells were resuspended in 100 µL of annexin-binding buffer and incubated with 5 µL of Alexa Fluor^®^ 488 Annexin V and 1 µL of PI (100 µg/mL) for 15 min at room temperature in the dark. After incubation, the cells were kept on ice and analyzed using a flow cytometer (CytoFLEX S, Beckman Coulter, Brea, CA, USA).

### 4.4. Colony Formation

HT-29 cells were seeded at 5 × 10^5^ cells per well in 6-well plates and allowed to attach for 24 h at 37 °C in 5% CO_2_. Cells were exposed to 0, 31.25, 62.5, 125, or 250 µM FEC for 24 h. The vehicle control received the same final DMSO concentration as the highest FEC dose. After treatment, the monolayers were washed twice with PBS, detached with 0.25% trypsin–EDTA, and resuspended in complete medium. Viable cells were counted by trypan blue exclusion using a hemocytometer, and 2 × 10^3^ viable cells per well were replated in fresh 6-well plates without FEC. Plates were incubated for 14 days, with medium changes every third day. Replating equal viable-cell numbers assessed colony-forming capacity among surviving cells after a defined exposure.

Colonies were fixed in 10% (*v*/*v*) formaldehyde for 45 min at room temperature, washed gently under running water, stained with 0.1% (*w*/*v*) crystal violet for 30 min and air-dried. Whole wells were photographed on a flat-bed scanner for the figure panels. Discrete clusters of 50 or more cells were scored as a colony under an inverted light microscope; clusters below this threshold, and colonies whose borders had merged, were excluded. Counting was performed independently by two operators blinded to the treatment identity, and the two counts were averaged. Each concentration was assayed in triplicate wells within an experiment, and the experiment was repeated three times independently.

Plating efficiency (%) = (number of colonies counted/number of cells seeded) × 100

Surviving fraction = plating efficiency of the treated group/plating efficiency of the vehicle control

Plating efficiency was calculated from the mean colony count [33]. The surviving fraction was normalized to the vehicle-control plating efficiency from the same experiment. Colony counts were expressed as mean ± SD of three independent experiments and compared with the vehicle control using one-way analysis of variance with Dunnett’s post hoc test; *p* < 0.05 was considered significant.

### 4.5. Cell Lysate Preparation and Western Blot Analysis

Total protein was extracted from untreated cells in the ten-cell-line panel and from HT-29 cells exposed for 24 h to FEC, FAC, VEC, or VAC at 25 µM, or to FEC at 0, 15, 30, or 60 µM. Cell pellets were resuspended in 1× RIPA buffer containing Triton X-100, PMSF, and protease inhibitors and sonicated on ice at 60% amplitude. Lysates were centrifuged at 12,500× *g* for 15 min at 4 °C, and supernatants were collected. Protein concentration was determined using a bicinchoninic acid assay. Samples and standards were mixed with working reagent prepared at a 50:1 ratio of reagents A and B, incubated for 30 min at room temperature, and measured at 562 nm. Equal amounts of total protein were loaded per lane. Samples were heated at 95 °C for 5 min, cooled, separated by SDS-PAGE, and transferred to PVDF membranes. Membranes were blocked for 1 h at room temperature in 5% nonfat dry milk in TBS-Tween (10 mM Tris base, 100 mM NaCl, and 0.1% Tween 20; pH 7.5). Primary antibodies against PPARγ and GAPDH were diluted 1:2000 in TBS-Tween and incubated with membranes overnight at 4 °C with shaking. Membranes were washed and incubated with secondary antibodies diluted 1:5000, then developed using enhanced chemiluminescence. Images were recorded using an iBright imaging system. For densitometry, lane boundaries were assigned by dividing the strip width equally, background was subtracted per column, and corrected intensities within each band window were integrated. PPARγ signals were normalized to GAPDH in the same lane and expressed relative to HT-29 for the cell-line panel or to the vehicle control for treatment experiments.

### 4.6. Association Between PPARγ Abundance and FEC Sensitivity

Each cell line contributed one paired observation: the basal PPARγ/GAPDH ratio relative to HT-29 and the mean residual MTT signal at 100 µM FEC from twelve wells across three independent experiments. The response at the highest common concentration was analyzed because most IC_50_ values were above the tested range and could not be treated as exact values. Pearson and Spearman correlations were calculated to examine sensitivity to the skewed distribution of PPARγ abundance. Leave-one-out analysis repeated the correlation after omitting each cell line in turn. Least-squares regression lines were fitted to the full panel and to the panel excluding HT-29. Analyses were performed in GraphPad Prism version 10.2.3.

### 4.7. Proteomics Analysis with Pathway Annotation

HT-29 cells were treated with FEC or vehicle for 24 h in four independent biological replicates per condition. Sample preparation followed a previous study with minor modifications [34]. Briefly, peptides were separated and analyzed by LC-MS/MS on an Orbitrap Exploris™ 480 mass spectrometer coupled to an UltiMate 3000 RSLCnano system fitted with a PepMap™ RSLC C18 column (2 µm, 100 Å, 75 µm × 25 cm; Thermo Fisher Scientific, Waltham, MA, USA). Raw files were processed in Proteome Discoverer using SEQUEST HT against the reviewed human UniProtKB database, with trypsin specificity, carbamidomethylation of cysteine as a fixed modification, and methionine oxidation and protein N-terminal acetylation as variable modifications [35]. Peptide and protein identifications were filtered to a 1% false discovery rate, and only master proteins were retained. Protein-level abundance ratios of FEC-treated to vehicle-treated samples, together with their adjusted *p* values, were taken from the Proteome Discoverer output and converted to log_2_ fold change and −log_10_ adjusted *p* value for downstream analysis. Proteins were considered differentially abundant when the adjusted *p* value was below 0.05 and the absolute log_2_ fold change was at least 1. Ratios reported at the software boundary values of 100 or 0.01, which arise when a protein is quantified in one condition but not the other, carry no meaningful magnitude and were therefore excluded from the fold change analysis and tabulated separately as presence or absence changes. Proteins without a calculable ratio were likewise excluded.

Functional protein sets were assembled from gene-symbol prefixes and Reactome an-notations supplied with the search output. Quantitative pathway methods such as Re-actomeGSA provide a broader context for abundance-based analysis [36]. The analysis used here was a separate competitive test. For each curated set, member-protein log_2_ fold changes were compared with those of all other quantified proteins using a two-sided Mann–Whitney U test. *p* values were adjusted across the tested sets using the Benjamini–Hochberg procedure. Sets with fewer than five quantified members were excluded. These exploratory tests summarized protein-set shifts and did not establish pathway activation or inhibition.

### 4.8. Statistical Analysis

Data are expressed as mean ± standard deviation. Biological replicates were independent experiments performed on different days with separately prepared cultures; technical replicates were wells within one experiment and were averaged before analysis. MTT data came from three independent experiments with four wells per concentration and were compared with the vehicle control by one-way analysis of variance (ANOVA) with Dunnett’s post hoc test. Western blot densitometry and colony counts came from three independent experiments and were analyzed in the same way. Proteomic data came from four biological replicates per condition. Analyses were performed in GraphPad Prism, and *p* < 0.05 was considered significant.

## 5. Conclusions

FEC showed moderate growth-inhibitory activity in HT-29 cells, with an IC_50_ of 42.8 ± 4.7 µM in the 24 h MTT screen. Comparisons across four hybrids and ten cancer cell lines identified dependence on both chemical structure and cellular context within the tested series. Reduced colony formation and a limited acute apoptotic response supported an antiproliferative component. Expression proteomics identified associated decreases in replication- and translation-related proteins and a modest collective increase in the abundance of endoplasmic reticulum protein-folding enzymes. PPARγ abundance did not consistently explain sensitivity across the panel, and FEC’s direct target remains undefined. These results provide a basis for further chemical optimization and mechanistic investigation. Ferulic acid alone was inactive in HT-29 cells, doxorubicin gave a comparable IC_50_ of 44.24 µM in the same assay, and FEC left non-cancerous Beas-2B cells above 92% viability at up to 100 µM. These findings provide preliminary evidence of differential sensitivity but do not establish broad safety or a therapeutic window.

## Figures and Tables

**Table 1:** IC_50_ values (µM) for three reference compounds and four coumarin hybrids across ten human cancer cell lines. Determined values are mean ± SD of three independent experiments. “>100” indicates that the MTT signal did not fall below 50% of the vehicle control at the highest screening concentration. “~100” denotes marginal inhibition by VEC in HT-29 cells, not a reproducibly determined IC_50_. Abbreviations: 7OH-Coumarin, 7-hydroxycoumarin; FEC, ferulic ester coumarin; FAC, ferulic amide coumarin; VEC, vanillic ester coumarin; VAC, vanillic amide coumarin.

Cell Line	Coumarin	7OH-Coumarin	Vanillic Acid	FEC	FAC	VEC	VAC
HT-29	>100	>100	>100	42.8 ± 4.7	>100	~100	>100
HCT116	>100	>100	>100	>100	>100	>100	>100
A549	>100	>100	>100	>100	>100	>100	>100
H460	>100	>100	>100	>100	>100	>100	>100
MCF-7	>100	>100	>100	>100	>100	>100	>100
MDA-MB-231	>100	>100	>100	>100	>100	>100	>100
HepG2	>100	>100	>100	>100	>100	>100	>100
Huh-7	>100	>100	>100	>100	>100	>100	>100
SW1088	>100	>100	>100	>100	>100	>100	>100
U-87MG	>100	>100	>100	>100	>100	>100	>100

## Data Availability

The datasets used and analyzed during the current study available from the corresponding author on reasonable request.

## Supplementary Materials

The following supporting information can be downloaded at: https://www.mdpi.com/article/10.3390/molecules31203729/s1, Table S1: Differential protein expression analysis of FEC-treated versus control samples. Figure S1: Cell viability after 24 h treatment, measured by MTT assay: HT-29 cells with ferulic acid (A) or doxorubicin (B), and non-cancerous Beas-2B cells with FEC (C), each at 0–100 µM. Values are mean ± SD of three independent experiments as a percentage of the vehicle control; one-way ANOVA with Dunnett’s post hoc test (* *p* < 0.05; **** *p* < 0.0001).
