# Supplementary material for: Coumarin–Ferulic Acid Hybrid Mediates HT-29-Selective Growth Inhibition and Proteomic Profiling
Source: Molecules. 2026 Oct 17;31(20):3729. doi: 10.3390/molecules31203729 (PMC13707454; doi:10.3390/molecules31203729)
Supplement: Supplementary file 1 [file molecules-31-03729-s001.zip › supplementary Figure S1.pdf]

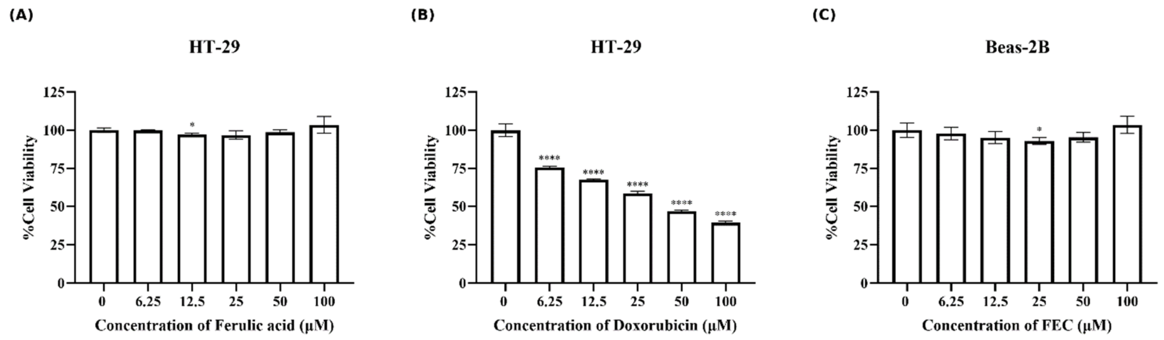

Figure S1: Cell viability after 24 h treatment, measured by MTT assay: HT-29 cells with ferulic acid (**A**) or doxorubicin (**B**), and non-cancerous Beas-2B cells with FEC (**C**), each at 0–100 μM. Values are mean ± SD of three independent experiments as a percentage of the vehicle control; one-way ANOVA with Dunnett's post hoc test (* $p < 0.05$; **** $p < 0.0001$).
